# A novel rapid visual detection assay for *Toxoplasma gondii* combining recombinase-aided amplification and lateral flow dipstick coupled with CRISPR-Cas13a fluorescence (RAA-Cas13a-LFD)

**DOI:** 10.1051/parasite/2022021

**Published:** 2022-04-14

**Authors:** Jinhong Zhao, Yuanyuan Li, Qiqi Xue, Zhiwei Zhu, Minghui Zou, Fang Fang

**Affiliations:** 1 Department of Medical Parasitology, Wannan Medical College Wuhu 241002 Anhui China; 2 Provincial Key Laboratory of Active Biological Macro-Molecules Wuhu 241002 Anhui China; 3 Department of Clinical Laboratory, The First Affiliated Hospital of Wannan Medical College Wuhu 241001 Anhui China

**Keywords:** *Toxoplasma gondii*, recombinase-aided amplification, lateral flow dipstick, CRISPR-Cas13a

## Abstract

Toxoplasmosis, a parasitic disease resulting from *Toxoplasma gondii* infection, remains prevalent worldwide, and causes great harm to immunodepressed patients, pregnant women and newborns. Although various molecular approaches to detect *T. gondii* infection are available, they are either costly or technically complex. This study aimed at developing a rapid visual detection assay using recombinase-aided amplification (RAA) and lateral flow dipstick (LFD) coupled with CRISPR-Cas13a fluorescence (RAA-Cas13a-LFD) to detect *T. gondii*. The RAA-Cas13a-LFD assay was performed in an incubator block at 37 °C within 2 h, and the amplification results were visualized and determined through LFD by the naked eye. The detection limit was 1 × 10^−6^ ng/μL by our developed RAA-Cas13a-LFD protocol, 100-fold higher than that by qPCR assay (1 × 10^−8^ ng/μL). No cross-reaction occurred either with the DNA of human blood or *Ascaris lumbricoides*, *Digramma interrupta*, *Entamoeba coli*, *Fasciola gigantica*, *Plasmodium vivax*, *Schistosoma japonicum*, *Taenia solium*, and *Trichinella spiralis*, and the positive rate by RAA-Cas13a-LFD assay was identical to that by qPCR assay (1.50% *vs.* 1.50%) in detecting *T. gondii* infection in the unknown blood samples obtained from clinical settings. Our findings demonstrate that this RAA-Cas13a-LFD assay is not only rapid, sensitive, and specific and allows direct visualization by the naked eye, but also eliminates sophisticated and costly equipment. More importantly, this technique can be applied to on-site surveillance of *T. gondii*.

## Abstract (fr)

La toxoplasmose, une maladie parasitaire résultant d’une infection à *Toxoplasma gondii*, reste répandue dans le monde et cause de graves dommages aux patients immunodéprimés, aux femmes enceintes et aux nouveau-nés. Bien que diverses approches moléculaires pour détecter l’infection à *T. gondii* soient disponibles, elles sont coûteuses ou techniquement complexes. Cette étude visait à développer un test de détection visuelle rapide utilisant une amplification assistée par recombinase et une bandelette réactive à flux latéral couplée à la fluorescence CRISPR-Cas13a (RAA-Cas13a-LFD) pour détecter *T. gondii*. Le test RAA-Cas13a-LFD a été effectué dans un bloc incubateur à 37 °C en 2 h, et les résultats d’amplification ont été visualisés et déterminés par LFD à l’œil nu. La limite de détection était de 1 × 10^−6^ ng/μL par notre protocole développé RAA-Cas13a-LFD, 100 fois plus élevée que celle du test qPCR (1 × 10^−8^ ng/μL). Aucune réaction croisée ne s’est produite ni avec l’ADN du sang humain ni avec *Ascaris lumbricoides*, *Digramma interrupta*, *Entamoeba coli*, *Fasciola gigantica*, *Plasmodium vivax*, *Schistosoma japonicum*, *Taenia solium* et *Trichinella spiralis*, et le taux de positivité par le test RAA-Cas13a-LFD était identique à celui par test qPCR (1,50 % contre 1,50 %) pour détecter l’infection à *T. gondii* dans les échantillons de sang inconnus obtenus en milieu clinique. Nos résultats démontrent que ce test RAA-Cas13a-LFD est non seulement rapide, sensible, spécifique et permet une visualisation directe à l’œil nu, mais élimine également les équipements sophistiqués et coûteux. Plus important encore, cette technique peut être appliquée à la surveillance sur place de *T. gondii*.

## Introduction

*Toxoplasma gondii*, an obligate intracellular protozoan parasite ubiquitous worldwide, is of medical and veterinary importance because it can cause toxoplasmosis that threatens warm-blooded animals and human health [21]. More than 30% of the world’s population is thought to have specific anti-*T. gondii* antibodies, though infection rates vary considerably by geographic region [26]. In China, antibody-positive rates for *T. gondii* were described to be 8.20% in the general population and 8.60% in pregnant women [11]. Characteristic symptoms do not always manifest in individuals with normal immunity, following infection with *T. gondii*, whereas premature delivery, abortion, teratosis, or stillbirth may occur in pregnant women regardless of apparent or recessive infection with this protozoan parasite, and even worse, newborns may develop ocular and neurological lesions [4, 5, 32]. For people with weakened immunity or immunodeficiency, such as patients with HIV/AIDS, organ transplant recipients, and tumor patients, infection by *T. gondii* can be associated with toxoplasmic encephalitis or even death [18, 37]. Therefore, it is crucial to develop a technique with high sensitivity and specificity for rapid and early diagnosis, prevention and control of toxoplasmosis.

Presently, approaches to diagnose of toxoplasmosis consist of pathogen detection, immunological techniques, and molecular biological methods [16, 20, 33]. Direct pathogenic diagnosis of *T. gondii* involves microscopic detection of tachyzoites or smear of tissue cysts. However, pathologic examination of tissues or strain isolation is primarily applied to diagnose animal infection, and less commonly human toxoplasmosis [32]. Nucleic acid and specific antibody assays of *T. gondii* are commonly used in clinical settings [9], particularly serum IgM/IgG antibody tests, such as enzyme-linked immunosorbent assay (ELISA), have been widely accepted as a primary screening technology for diagnosis of toxoplasmosis. However, this protocol may fail to detect specific anti-*T. gondii* antibodies during the active infection phase because of a gap of several weeks between antibody production and development of parasitemia. In recent decades, diagnosis of *T. gondii* on a molecular basis, including PCR, nested PCR, and real-time PCR (qPCR) assays, has been in place. These PCR-based amplification techniques have demonstrated optimal sensitivity and specificity in early diagnosis of toxoplasmosis [22], but they are limited in wider clinical application due to complex procedures in detection, costly test equipment, requirements for qualified technical personnel, and so on.

To date, some new molecular biology technologies operating without thermocycling but at constant incubation temperature have been described, which can greatly reduce the reaction time and complexity of nucleic acid amplifying procedures. For instance, loop-mediated isothermal amplification (LAMP) performed in only a heating block, can achieve constant temperature, which is superior to an expensive thermocycler [32]. Unfortunately, the LAMP technique is prone to aerosol contamination that potentially results in false-positive findings [32]. Recombinase-aided amplification (RAA) assay is a newly developed technology for nucleic acid amplification, and involves only three major proteins, i.e., recombinase UvsX, single strand DNA binding protein (SSB), and DNA polymerase at a constant temperature [35]. In addition, RAA assay can be completed in a short reaction time (30 min) merely with a simple thermostatic device (constant temperature 37–42 °C) [31], and the amplified DNA is detected by agarose gel electrophoresis (AGE), real-time fluorescence, and lateral flow dipstick (LFD) [36]. Among these optimizations, the LFD method can also detect proteins and nucleic acids, and the results are readily interpretable [17]. Meanwhile, RAA-LFD has also been applied to the detection of microbes, such as Newcastle disease virus [28], dengue virus [31], avian infectious laryngotracheitis virus [29], and novel coronavirus [38], and even to identifying the genetic sex of *Cynoglossus semilaevis* [36].

The CRISPR-Cas system has shown great potential as a practical and powerful diagnostic tool for its ability of trans-cleavage to accurately recognize and cleave specific nucleic acid targets [1, 2, 19]. The Cas nucleases contain RNA-guided RNases (Cas13a and Cas13b) and RNA-guided DNases (Cas12a, Cas12b and Cas14), which are able to exhibit non-specific trans-cleavage activity after binding to their specific targets [7, 8]. Cas12a and Cas13 are the main nucleases for the development of CRISPR-Cas-based nucleic acid detection with high sensitivity and specificity to recognize a nucleic acid target. Also, CRISPR-Cas is developed to be jointly used with isothermal amplification, such as RAA, to enhance diagnostic accuracy [30, 39]. In a study of combined use of RAA with CRISPR-Cas12a, *T. gondii* was successfully detected in nearly an hour at body temperature, and the detection was more sensitive than conventional PCR assay [19]. CRISPR-Cas13a also exhibited higher sensitivity and good specificity in detecting DNA sequences with DNA endonuclease-targeted CRISPR trans-reporter [6]. Methods such as SHERLOCK (specific high-sensitivity enzymatic reporter unlocking), which typically uses target amplification followed by CRISPR-mediated nucleic acid detection, have been used to detect SARS-CoV-2 [12]. However, few reports are available on the use of CRISPR-Cas13a systems for detection of *T. gondii*.

For the diagnosis of toxoplasmosis, a series of nucleic acid amplification assays targeting the B1 gene or 529 bp repeat sequences, internal transcriptional spacer sequences (ITS-1), and 18S rDNA sequences have been established. The 529 bp repeat sequences show better sensitivity and specificity during diagnosis of toxoplasmosis [23]. According to the literature, RAA amplification and LFD detection coupled with CRISPR-Cas13a fluorescence assay used for *T. gondii* detection has not been reported so far. In this study, we designed a crRNA probe with the Cas13a-based detection system in combination with RAA and LFD by targeting the repeated 529 bp element of *T. gondii* for easy visualization, and validated the established RAA-Cas13a-LFD assay by detecting *T. gondii* in genomic DNA extracted from clinical blood samples.

## Materials and methods

### Ethics approval

All aspects of the study were performed in accordance with national ethics regulations, and approved by the Institutional Review Board (IRB) of Wannan Medical College, Anhui Province, China.

### Strains and clinical samples

*Toxoplasma gondii* tachyzoites (RH strain) were preserved in our laboratory, and aseptically cultured *in vitro* by serial passages in Vero cells, as per procedures described previously [14]. A total of 267 blood samples were obtained from the First Affiliated Hospital of Wannan Medical College between December 2018 and November 2020. Sampling of the blood was conducted by qualified laboratory technicians from the hospital to prevent unnecessary risks of exposure. The age range of patients (*n* = 267) who provided blood samples was 18–60 years. Of these patients, 137 were men and 130 women, and 65 had a malignant tumor, 62 rheumatoid arthritis, 60 pathological pregnancy, and another 80 were healthy volunteers. No participants had a history of medical examination for toxoplasma. Two aliquots of blood were taken from each patient, and maintained respectively in a tube with anticoagulant (containing 10 μL 0.5 μMol/L EDTA) and another without anticoagulant. The blood in the non-anticoagulant tube was centrifuged, and the serum was aseptically separated for serology via electrochemiluminescence (ECL) assay (Roche, Switzerland, 04618858190 V11). Blood from the anticoagulant tubes was used to extract genomic DNA.

### DNA extraction

A DNeasy Blood & Tissue Kit (Tiangen Biotech Beijing Co., Ltd., China) was used to extract genomic DNA from the blood samples, according to the manufacturer’s instructions. All DNA samples were stored at −20 °C until subsequent analysis. In view of the low amount of circulating DNA in these blood samples whose status infection was unknown, each sample was divided into three portions, and repeatedly eluted with 50 μL elution solution to ensure the DNA yield and detection rate. Then, each blood replicate was tested, and accepted as infection in any portion of the blood showing a positive result.

### Construction of standard recombinant plasmids

The 529 bp repeated element (GenBank AF146527) is repeated between 200 and 300 copies in the genome of *T. gondii*, and therefore considered the potential optimal target for *T. gondii* [10], because it can generate higher sensitivity and specificity compared to target sequences B1 and 18S rDNA in molecular diagnosis of toxoplasmosis [24]. In this study, we initially amplified a 529 bp fragment in the *T. gondii* tachyzoites, and had PCR products electrophoresed, excised, purified, and then cloned into the pMD^TM^ 19-T vector (Takara). The positive clones were sequenced directionally (ABI 3730), and the positive recombinant plasmids of *T. gondii*, previously kept in our laboratory (Wannan Medical College, China), were used as standard nucleic acids to quantify the sequences with a NanoDrop ND1000 spectrophotometer. Then, the recombinant plasmids were prepared with a dilution range of 1 ng/μL–1 × 10^−9^ ng/μL as a standard control and stored at −20 °C for further use.

### Design of primer and crRNA probe

In order to facilitate successful RAA amplification depending on ideal primers for the target gene, we designed five pairs of specific oligonucleotide primers targeting the 529 bp repeated element of *T. gondii* by using online primer design software (https://www.ncbi.nlm.nih.gov/tools/primer-blast). Annealing temperature was set at 54–67 °C for the primer with a length of 30–35 bp, and the amplicon size was 100–300 bp (Table 1). Specific crRNA probes were designed (https://www.ncbi.nlm.nih.gov/tools/primer-blast) to target the specific primer sequence fragment (Table 1). The DNA probes were transcribed into RNA, according to the procedures described in the user instructions of the HiScribe T7 Quick High Yield RNA Synthesis Kit (New England Biolabs, Ipswich, MA, USA). The 5′ end accessory T7 RNA polymerase promoter sequence was considered during probe design. The transcript (crRNA probe) was purified using RNAXP magnetic beads, and product concentration was determined by Qubit fluorometric quantitation (Thermo Fisher Scientific, Waltham, MA, USA). All primers were synthesized by TsingKe Biotech (Beijing TsingKe Biotechnology, Beijing, China).

**Table 1:** Primer sequences for RAA-Cas13a-LFD assay.

Primers	Sequences (5′ to 3′)	Amplicon sizes (bp)	Description
TOX-F1	ACTACAGACGCGATGCCGCTCCTCC	283	Candidates for RAA primers screened in this study. TOX-F1/TOX-R1 pair was used for follow-up experimental tests.
TOX-R1	TGTCTCCCTCGCCCTCTTCTCCACT		
TOX-F2	ACACCGGAATGCGATCCAGACGAGA	126	
TOX-R2	GTCCAAGCCTCCGACTCTGTCTCCC		
TOX-F3	ATATCAGGACTGTAGATGAAGGCGAGGGT	176	
TOX-R3	CGTCTCGTCTGGATCGCATTCCGGTGTCT		
TOX-F4	TAGATGAAGGCGAGGGTGAGGATGAGGGG	121	
TOX-R4	CTCCAGGAAAAGCAGCCAAGCCGGAAACA		
TOX-F5	AAGATGTTTCCGGCTTGGCTGCTTTTCCTG	162	
TOX-R5	CCGACTCTGTCTCCCTCGCCCTCTTCTCCA		
T7 promoter	GAAATTAATACGACTCACTATAGGG		Promoting the transcription of amplified DNA to RNA.
Probe 1	CGUCUCGUCUGGAUCGCAUUCCGGUGUC		Expected sequences of guide RNA obtained from *in vitro* transcription. Probel was used for follow-up experimental tests.
Probe 2–5-a	UUCUCUCCGCCAUCACCACGAGGAAAGC		
Probe 2–5-b	CAAUUCUCUCCGCCAUCACCACGAGGAA		
Probe 3–4-a	CUCCGACUCUCGUCGCUUCCCAACCACG		
Probe 3–4-b	CCGACUCUCGUCGCUUCCCAACCACGCC		
Lateral flow reporter	FAM/mArArUrGrGrCmAmArArUrGrGrCmA/Bio		Trans-cleavage reporter for Cas13a.

### Establishment of the RAA assay

Basic RAA reactions were performed using Basic RAA kits (Anhui Microanaly Genetech, Hefei, China). The reaction buffer was premixed by formula as: 25 μL of rehydration buffer, upstream primers (10 μMol/L) 2 μL, downstream primers (10 μMol/L) 2 μL, 25 × SYBR Green I 1 μL, recombinant plasmids (1 ng/μL) 2 μL, MgOAc solution (280 nMol/L) 2.5 μL, and 17.5 μL of nuclease-free water. The reaction tube aforementioned was then placed in a fluorescent quantitative PCR instrument to amplify for 40 min at 37 °C. The amplified products were analyzed with a Qsep100 biological analyzer (BIOptic, Taiwan) by collecting the fluorescence.

### Establishment of the RAA-Cas13a-LFD assay

**Figure 1:**
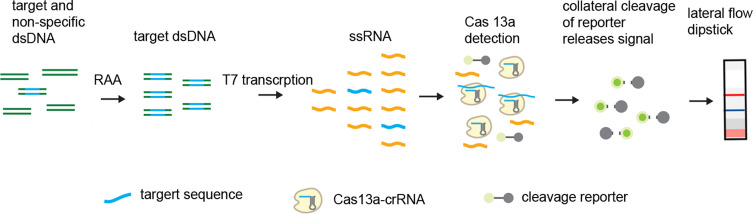
Schematic for *T. gondii* detection with the RAA-Cas13a-LFD assay. The procedures include amplification of target DNA with RAA; T7 RNA polymerase transcription of amplified DNA to RNA; target recognition of the target RNA with Cas13a-crRNA, collateral cleavage of fluorophore-quencher reporter (i.e., lateral flow reporter) and release fluorophore signal; visualization of the generated fluorescence signal by lateral flow dipstick.

Targeted detection was performed using gene editing technology based on nucleic acid amplification and Cas13a-mediated collateral cleavage of a reporter RNA, allowing for real-time detection of the target. The 529 bp fragment of *T. gondii* was amplified by RAA. T7 RNA polymerase transcription amplified DNA to RNA and target RNA was detected by Cas13a collateral RNA cleavage-mediated release of reporter signal. The fluorescence reporter signal was detected by LFD, an endpoint assay with lateral flow strips exposed to the reaction mixture following incubation (Fig. 1).

The RAA-Cas13a-LFD reaction system was a totally mixed volume of 8 μL, consisting of LwCas13a nuclease (45 nMol/L) (Anhui Microanaly Genetech, Hefei, China), lateral flow reporter molecule (125 nMol/L) (Integrated DNA Technologies, Coralville, IA, USA), RNase inhibitor (1 μL) (New England Biolabs), ATP (1 mM), GTP (1 mM), UTP (1 mM), CTP (1 mM), and T7 polymerase (0.6 μL), into which 1 μL of crRNA probe (30 ng/μL) and 1 μL of RAA products were added, mixed thoroughly, and incubated at 37 °C for 40 min. Then 20 μL of diluent buffer was added into the 10 μL of the reaction and fully mixed. The LFD was inserted into the mixture for 3–5 min and the results were recorded up to exposure of the positive control line.

### Interpretation of RAA-Cas13a-LFD assay results

**Figure 2:**
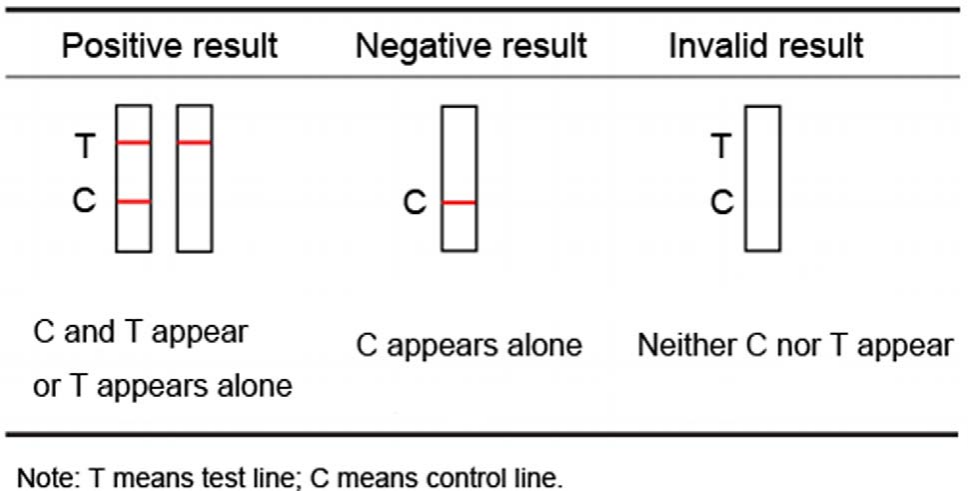
Interpretation of RAA-Cas13a-LFD assay results. Positive findings: both test line and control line displayed or only the test line developed. Negative findings: only control line visualized. Invalid results: Neither test line nor control line appeared.

Cas13a, with trans-cleavage activity, can cleave single stranded RNA (ssRNA) in a non-specific fashion. This activity makes Cas13a-based detection possible to apply to lateral flow detection with the FAM-ssRNA-Biotin reporter (FB reporter) [7, 25]. Without trans-cleavage, the FB reporter will remain intact, and its biotinylated end can be captured by streptavidin at the control line. Anti-FAM antibody conjugated gold nanoparticles can then bind the exposed FAM moiety, resulting in the color deposit on the control line. The tested results are considered to be negative if just the control band (red band) appears which indicates failed amplification of the target gene fragment in the sample (Fig. 2). On the other hand, if the FB reporter undergoes trans-cleavage by Cas13a, biotin instead of FAM will harbor in a portion of the reporter molecules. As a result, fewer FAM ends will display at the control line, yet more antibody-conjugated gold nanoparticles tend to collect on the test line. The test is then justified to be positive upon occurring of both the test line and control line or single test line which indicates that the target gene of *T. gondii* was successfully detected in the samples.

### Evaluation of RAA-Cas13a-LFD specificity and sensitivity

To determine the analytical specificity of the RAA-Cas13a-LFD assay developed in this study, the nucleic acids of human blood, *Ascaris lumbricoides*, *Digramma interrupta*, *Entamoeba coli*, *Fasciola gigantica*, *Plasmodium vivax*, *Schistosoma japonicum*, *Taenia solium*, and *Trichinella spiralis* were included as templates for RAA reaction. RAA-Cas13a-LFD assay sensitivity was assessed using 10-fold serial dilutions of the recombinant plasmids ranging from 1 ng/μL to 1 × 10^−9^ ng/μL. All experiments were carried out in duplicate by using recombinant plasmid as positive control, and ddH_2_O as negative control. Different RAA products were directly analyzed by lateral flow dipsticks.

The analytical specificity and sensitivity of the RAA-Cas13a-LFD assay were validated via real-time PCR (qPCR) by using forward, reverse and probe primers targeting the same 529 bp repeated fragment. The sequences of the primers were AGACGAGACGACGCTTTCC, GCATCTGGATTCCTCTCCTACC, and CGTCCAAGCCTCCGACTCTGTCTC, respectively. qPCR was performed in 20 μL reaction volume that was composed of 10 μL of 2 × Master Mix (Roche, Switzerland), 10 μM primer for each forward and reverse primer, 10 μM probe primer, and 2 μL template. Amplification was performed as follows: initial denaturation at 95 °C for 30 s, followed by 40 cycles of denaturation at 95 °C for 10 s; primer annealing at 60 °C for 30 s, and dissociation at 95 °C for 15 s, 60 °C for 60 s, and 95 °C for 15 s. The qPCR products were visualized according to an amplification curve generated by a LightCycler 96 qPCR instrument (Roche, Switzerland).

### RAA-Cas13a-LFD assay used in clinical samples

The previously established RAA-Cas13a-LFD protocol was used to detect *T. gondii* in blood samples collected from patients and healthy volunteers from a clinical laboratory. A total of 267 blood samples were simultaneously detected with the serology ECL assay, qPCR, and RAA-Cas13a-LFD assay for *T. gondii*. In order to evaluate the validity of the established RAA-Cas13a-LFD assay, a qPCR assay was additionally performed to amplify the 529 bp fragment of *T. gondii* in all blood samples.

## Results

### Optimization of RAA primers

**Figure 3:**
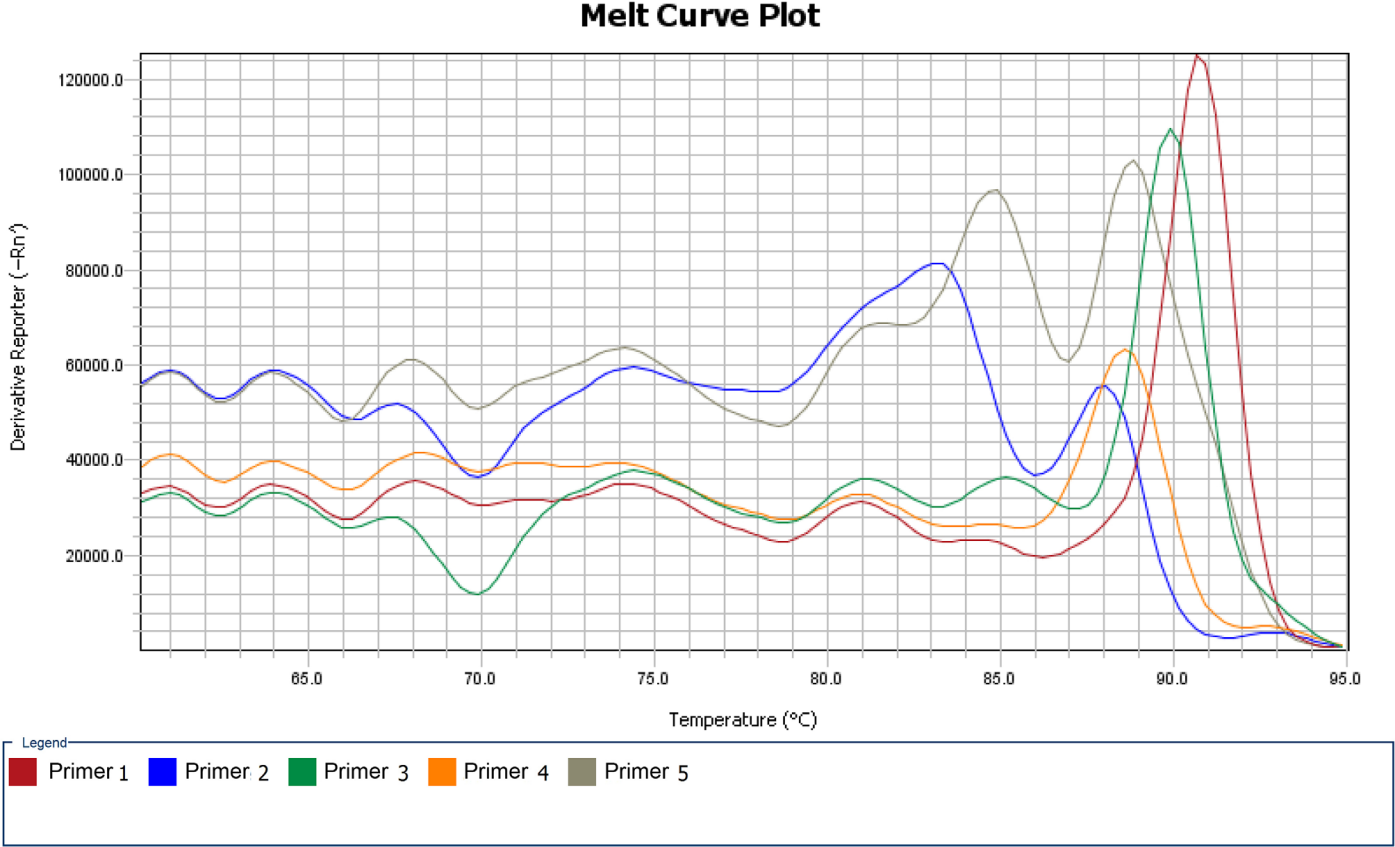
Melting curves for the five primer pairs. Primer pairs 1 and 3 had a single high peak, and were highly specifically amplified. Impure peaks developed in primer pairs 2 and 5, resulting in nonspecific amplification. Primer 4 had a single low peak, indicating poor amplification efficiency.

Five pairs of primers were designed for the 529 bp repeated fragment region in compliance with RAA nucleic acid amplification (Table 1). The products were initially amplified by RAA nucleic acid at 37 °C for 40 min, and then screened by fluorescence and capillary electrophoresis. According to the primer melting curves (Fig. 3), the primer pairs 1 and 3 had a single high peak, yet demonstrated no nonspecific amplification. The fragment size of the amplified products was about 283 bp and 176 bp, respectively for primer pairs 1 and 3, and consistent with the expected target fragment. In addition, the total amount was relatively higher in the products of primer pairs 1 and 3 (Fig. 3, Table 2). Impurity peaks developed in the melting curve of primer pairs 2 and 5, which indicated nonspecific amplification and low total amount of amplified products (Fig. 3, Table 2). Although the primer dissolution curve of primer 4 exhibited one single peak, the total amount of product being low explained poor amplification efficiency by primer 4 (Fig. 3, Table 2). Therefore, primer pairs 1 and 3 were efficient candidates for optimizing the probe, based on the results of the melting curve and the peak of the amplified product.

**Table 2:** Amplified products of the 5 pairs of primers by Qsep100 bioanalyzer test.

Primer pair	Size of main peak (bp)	Total amount of target product (ng)	Total amount of nonspecific amplification products (ng)	Target product ratio (%)
Primer pair 1	283	990	0	100
Primer pair 2	126	72	90	44
Primer pair 3	176	560	0	100
Primer pair 4	121	150	0	100
Primer pair 5	162	160	164	49

### Optimization of crRNA probe primers

**Figure 4:**
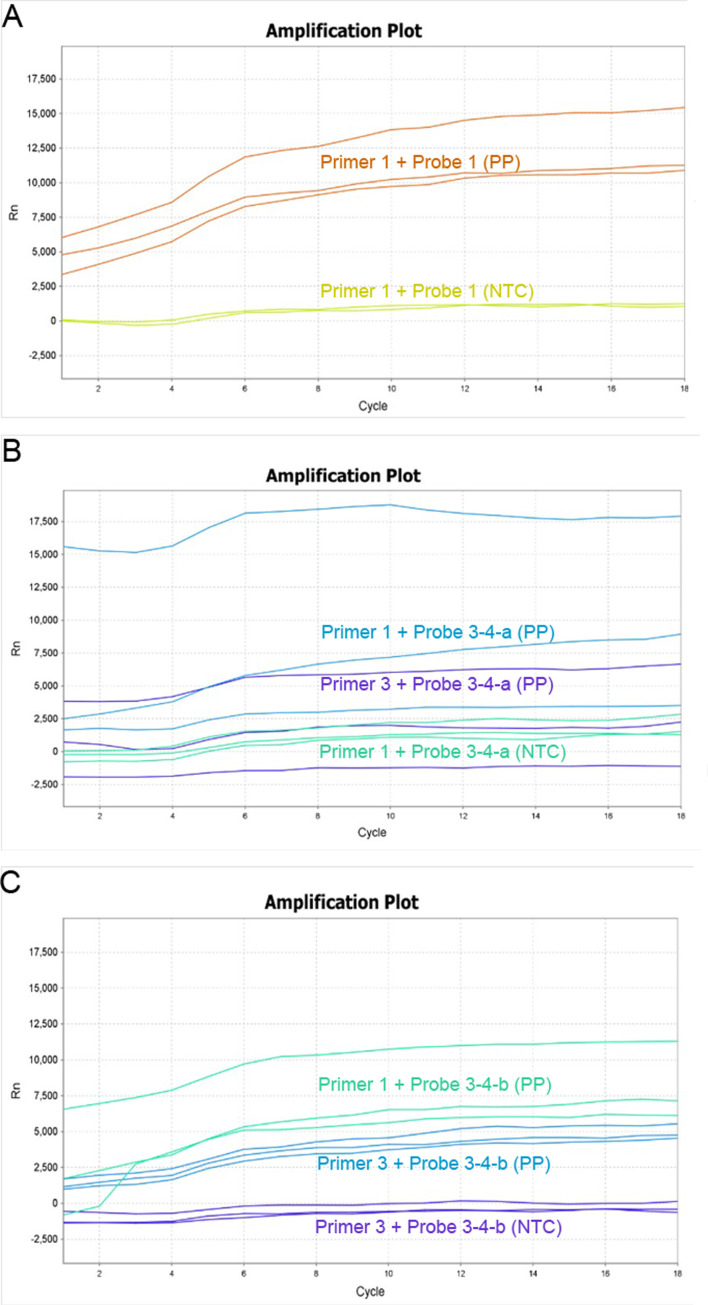
Fluorescence amplification curve of primer and probe combination. (A) The fluorescence amplification curve of Primer 1 + Probe 1 is very high in the positive plasmid control of *T. gondii* for three repeats; (B) The fluorescence curve of Primer 1 + Probe 3–4-a and Primer 3 + Probe 3–4-a indicates no significant amplification, and the consistency is poor for three repeats; (C) The fluorescence curve of Primer 1 + Probe 3–4-b and Primer 3 + Probe 3–4-b also shows no significant amplification, and the amplification consistency of Primer 1 + Probe 3–4-b is poor for the three repeats. PP: positive plasmid control; NTC: negative control.

After isothermally amplifying the positive plasmid using RAA, the fluorescence method was used to optimize the combination of primers and probes. The amplification efficiency of primer-probe combinations was evaluated by a fluorescence amplification curve (Fig. 4A). The fluorescence value of the positive plasmid group based on the Primer 1 + Probe 1 combination was significant different compared to the negative control (NTC) group by the curve, which indicated positive detection results and satisfactory amplification efficiencies. There was also significant amplification based on the Primer 1 + Probe 3–4-a and Primer 3 + Probe 3–4-a combinations, whereas the consistency was poor (Fig. 4B). The amplification curve was in poor consistency for Primer 1 + Probe 3–4-b, and the fluorescence value of Primer 3 + Probe 3–4-b was not significantly different compared with that of the NTC group; the amplification efficiency was markedly lower (Fig. 4C). In summary, the Primer 1 + Probe 1 combination had the best amplification efficiency, and was thus used in the subsequent RAA-Cas13a-LFD experiments.

### Optimization of the RAA-Cas13a-LFD assay conditions

**Figure 5:**
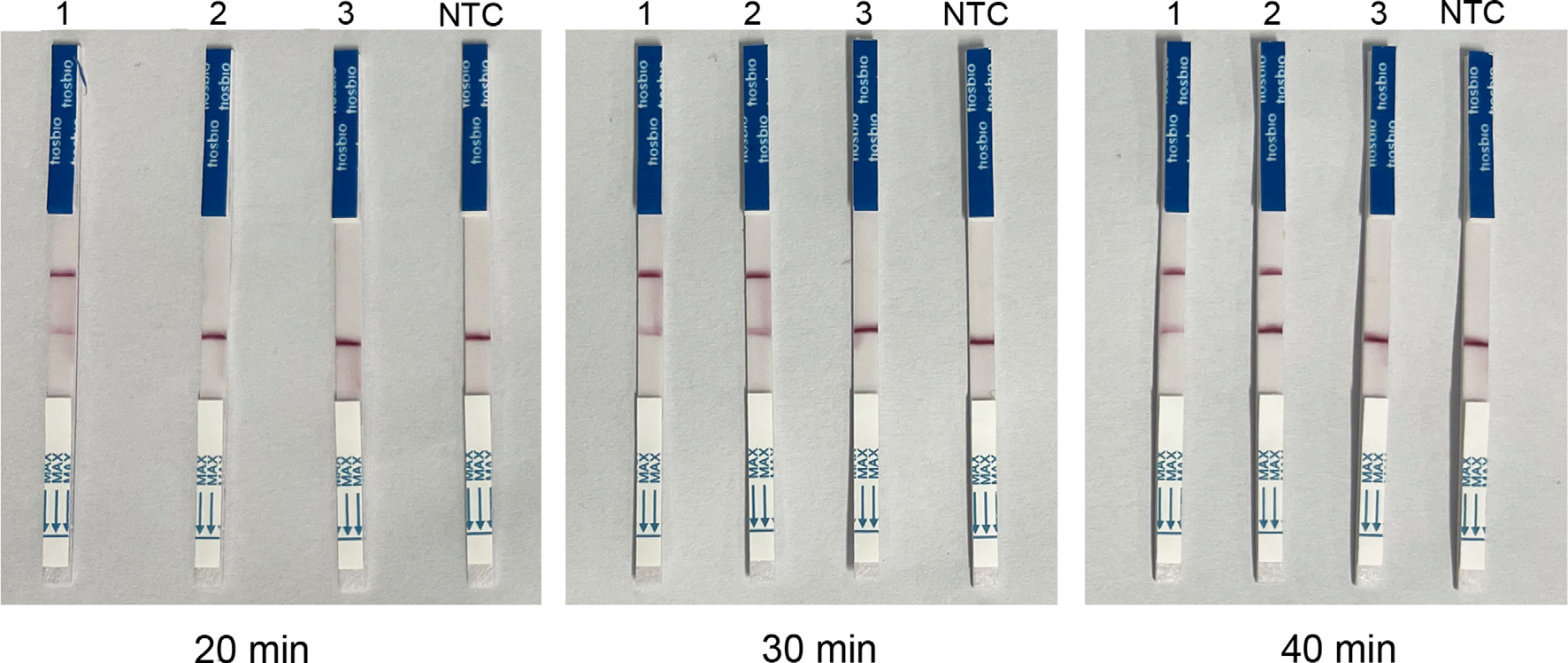
Optimization result of RAA-Cas13a-LFD amplification time. Strip: 1. 1 × 10^−5^ ng/μL; 2. 1 × 10^−6^ ng/μL; 3. 1 × 10^−7^ ng/μL; NTC: negative control (ddH_2_O).

The ideal time for the RAA-Cas13a-LFD reaction was evaluated. RAA basic reactions were conducted at 37 °C for 40 min in a heating block. The results showed that the target DNA was successfully amplified. Body temperature of 37 °C was decided as the optimal RAA-Cas13a-LFD reaction condition. Plasmid at concentrations of 1 × 10^−5^ ng/μL, 1 × 10^−6^ ng/μL, and 1 × 10^−7^ ng/μL was used as individual templates, respectively and amplified for 20, 30 and 40 min. The results showed that the detection limit of plasmid was at concentrations of 1 × 10^−5^ ng/μL when the RAA-Cas13a-LFD reaction was amplified for 20 min, and by 30 and 40 min of RAA-Cas13a-LFD reaction, the detection limit of plasmid concentrations was 1 × 10^−6^ ng/μL (Fig. 5). The detection limit of amplification yield did not change with the time from 30 min to 40 min. Therefore, 30 min was set as the RAA-Cas13a-LFD reaction time in this study.

### Sensitivity of the established RAA-Cas13a-LFD assay

**Figure 6:**
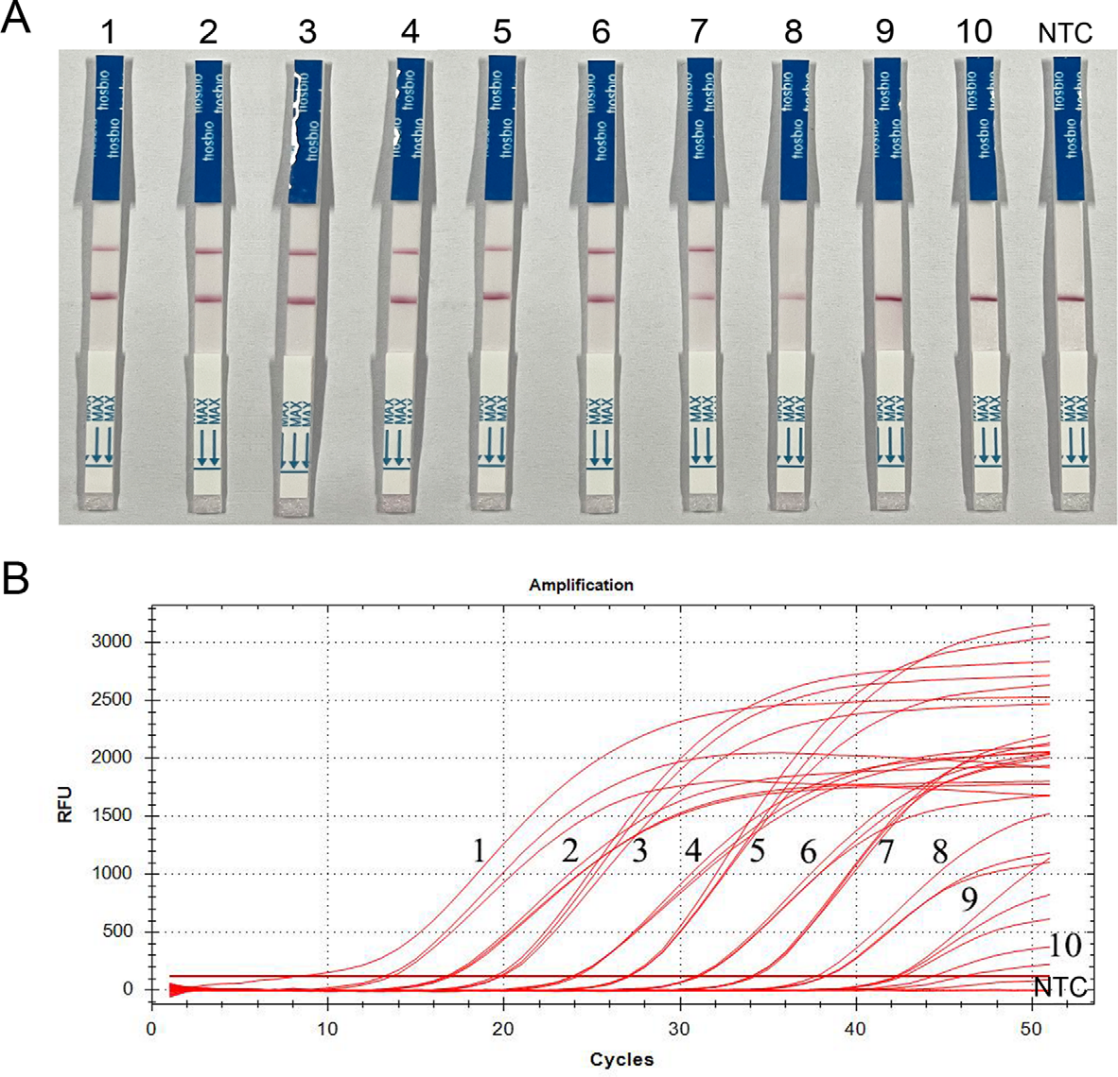
Sensitivity of *T. gondii* detection by RAA-Cas13a-LFD and qPCR assay. (A) Visual inspection of RAA-Cas13a-LFD. (B) Curves for qPCR. Strip and curves: 1–10: plasmid copy number diluted to 1 ng/μL–1 × 10^−9^ ng/μL, respectively; NTC: negative control (ddH_2_O).

Sensitivity of the RAA-Cas13a-LFD assay was determined by using serial 10-fold dilutions of a positive control template (positive recombinant plasmid of *T. gondii*). These dilutions ranged from 1 ng/μL to 1 × 10^−9^ ng/μL, with ddH_2_O included as a negative control. The reaction was amplified for 30 min, and the lowest detection concentration of the RAA-Cas13a-LFD was determined. The test lines visibly occurred at the concentration of recombinant plasmids being > 1×10^−6^ ng/μL (Fig. 6A). Further verification by qPCR showed that the minimum detection limit of qPCR was 1 × 10^−8^ ng/μL (Fig. 6B). These results indicated that the limit of the RAA-Cas13a-LFD assay for *T. gondii* was below qPCR in sensitivity.

### Specificity of the established RAA-Cas13a-LFD assay

**Figure 7:**
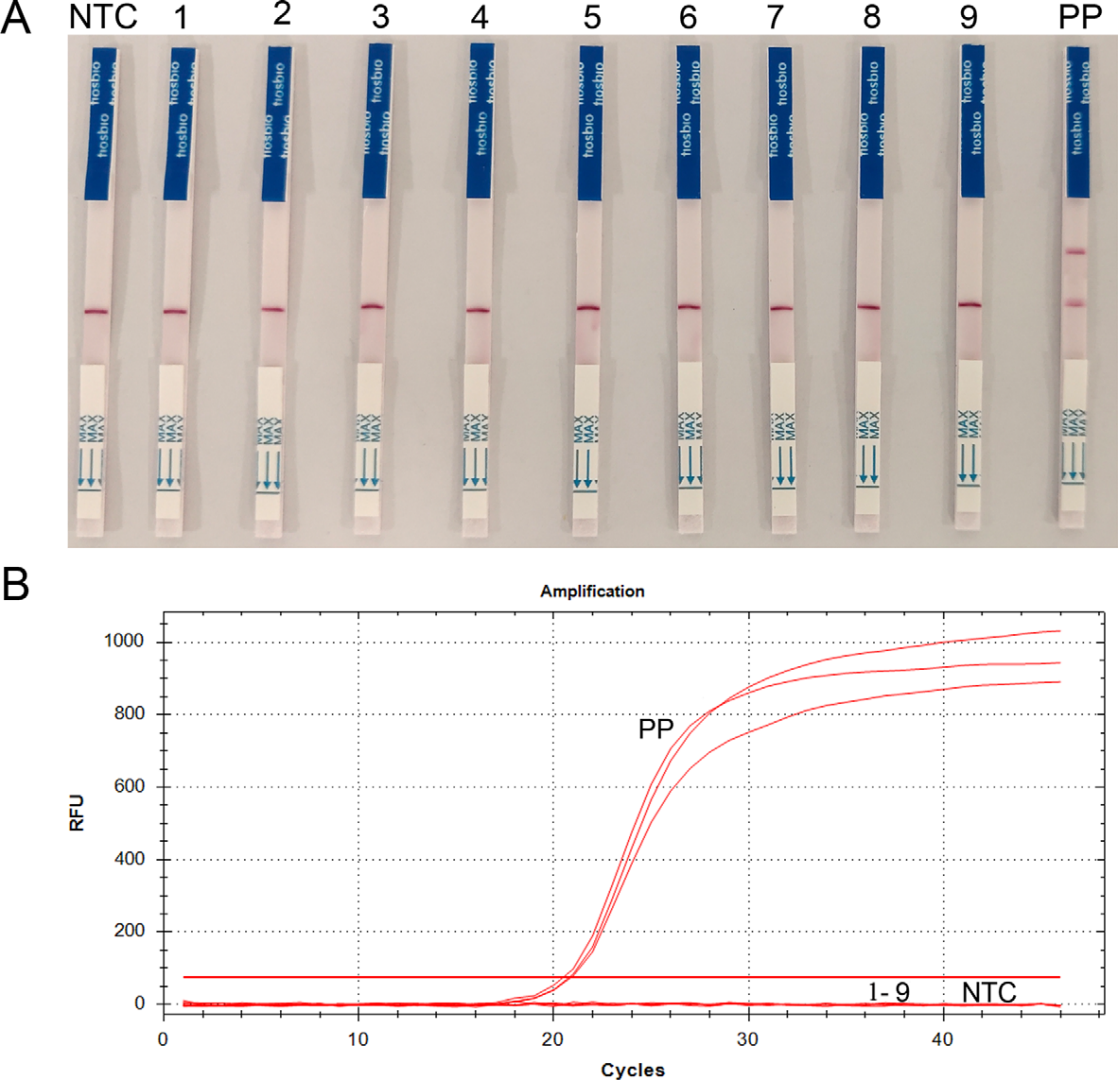
Specificity of *T. gondii* detection by RAA-LFD and qPCR detection. (A) Visual inspection of RAA-Cas13a-LFD. (B) Curves for qPCR. Strip and curves: 1. Human blood; 2. *Ascaris lumbricoides*; 3. *Digramma interrupta*; 4. *Entamoeba coli*; 5. *Fasciola gigantica*; 6. *Plasmodium vivax*; 7. *Schistosoma japonicum*; 8. *Taenia solium*; 9. *Trichinella spiralis*; PP: Positive plasmid of *T. gondii*; NTC: negative control (ddH_2_O).

Specificity was verified for and compared between RAA-Cas13a-LFD and qPCR by using genomic DNA samples from human blood, *Ascaris lumbricoides*, *Digramma interrupta*, *Entamoeba coli*, *Fasciola gigantica*, *Plasmodium vivax*, *Schistosoma japonicum*, *Taenia solium*, and *Trichinella spiralis*. *Toxoplasma gondii-*positive plasmid was used as positive control, and ddH_2_O as negative control. Only *T. gondii*-positive plasmids were amplified under both assays, and generated visible test bands (RAA-Cas13a-LFD) and amplification curves (qPCR). Amplification was blank under other DNA templates (Figs. 7A and 7B), which demonstrated that the established RAA-Cas13a-LFD method was more specific to *T. gondii* detection.

### Assessment of the RAA-Cas13a-LFD assay on clinical samples

**Figure 8:**
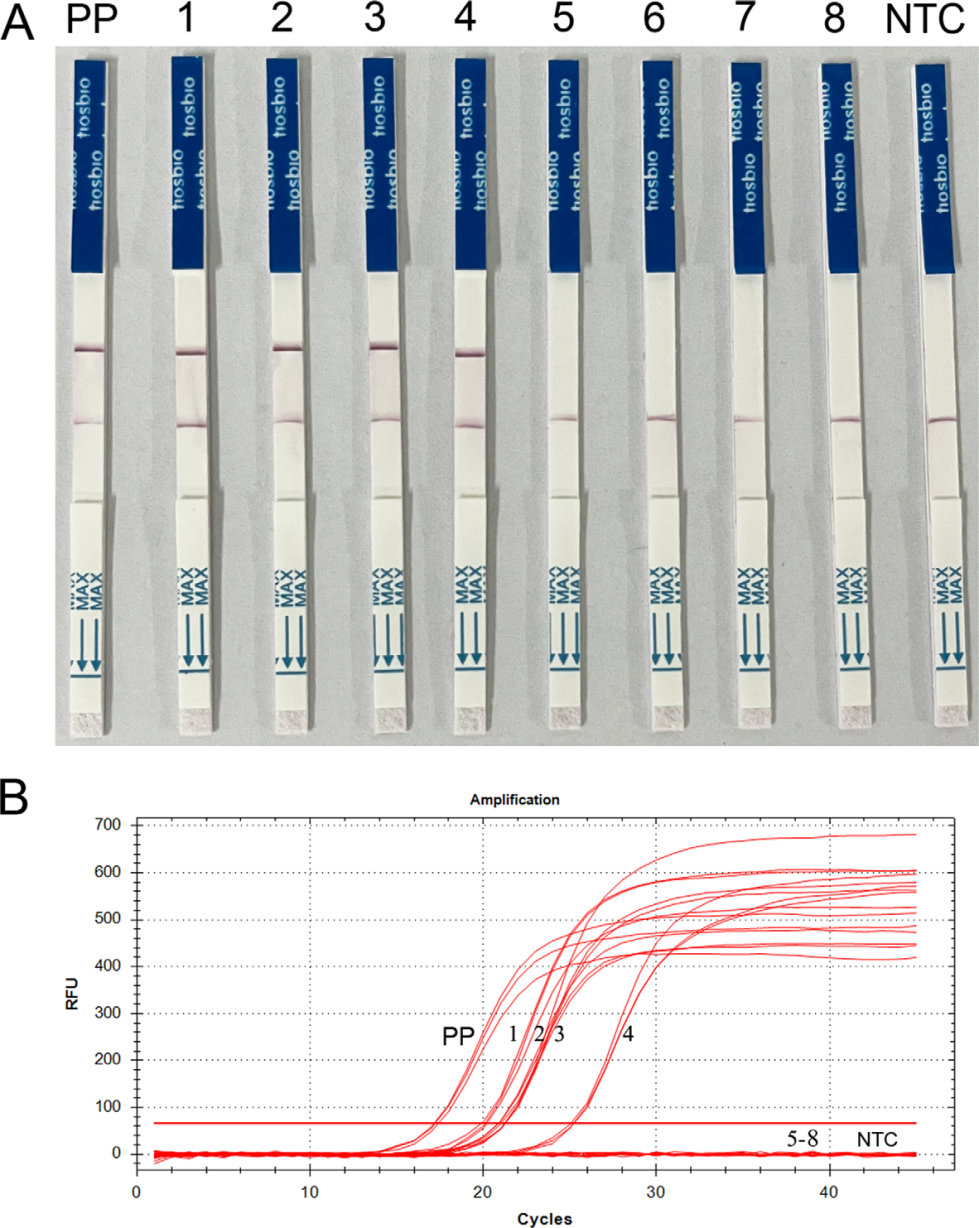
*Toxoplasma gondii* detected in the blood samples via RAA-Cas13a-LFD and qPCR. (A) Visual inspection of RAA-Cas13a-LFD; (B) Curves for qPCR. Strip and curves: 1–4: positive sample; 5–8: negative samples; PP: Positive plasmid of *T. gondii*; NTC: negative control (ddH_2_O).

In order to test the feasibility to detect *T. gondii* of the RAA-Cas13a-LFD assay on human samples, a total of 267 clinical blood samples were collected from patients and volunteers, and tested using ECL, qPCR, and our established RAA-Cas13a-LFD to validate whether *T. gondii* infection was present in the unknown samples. The positive rates of *T. gondii* in the blood samples varied among the three assays (Table 3). In total, 4 out of the 267 blood DNA samples were found to be positive for *T. gondii* detected by RAA-Cas13a-LFD and qPCR targeting the 529 bp sequence, which indicated compatibility of the two assays (Fig. 8, Table 3). Three samples were positive for *T. gondii* by ECL-IgM antibody detection. RAA-Cas13a-LFD (1.50%, 4/267) and qPCR (1.50%, 4/267) generated relatively higher positive rates than ECL-IgM (1.12%, 3/267) (Table 3). These results indicate that the detection of *T. gondii* in human samples is possible with the established RAA-Cas13a-LFD assay. A more comprehensive analysis including more positive clinical samples is needed to fully validate the clinical performances, in particular the sensitivity, in routine practice and also to compare the clinical performance of the established RAA-Cas13a-LFD assay with that of qPCR.

**Table 3:** Comparison of the results by ECL, qPCR and RAA-Cas13a-LFD assay for detection of *T. gondii* in the blood samples.

Test methods	Blood samples (*n* = 267)					
	Positive findings for patients with malignant tumor (*n* = 65)	Positive findings for patients with rheumatoid arthritis (*n* = 62)	Positive findings for patients with pathological pregnancy (*n* = 60)	Positive findings for healthy volunteers (*n* = 80)	Total positive rate	Total negative rate
ECL						
IgG	12	10	8	4	34 (12.73%)	233 (87.27%)
IgM	1	1	1	0	3 (1.12%)	264 (98.88%)
qPCR	2	1	1	0	4 (1.50%)	263 (98.50%)
RAA-Cas13a-LFD	2	1	1	0	4 (1.50%)	263 (98.50%)

## Discussion

Thus far, several nucleic acid-based detection platforms have been developed for *T. gondii*, including traditional PCR, nested PCR, and qPCR. Although these methods possess better sensitivity, they have certain deficiencies, such as availability of complex and costly testing devices and inability to satisfy point-of-need application due to complexity of testing procedures [16]. LAMP is characterized by a streamlined protocol and an exemplary detection limit of one copy of *T. gondii* DNA per reaction, but spurious amplification can arise, and the method is prone to aerosol pollution [27]. RAA-LFD is a combined isothermal amplification of the target gene through labeling the RAA probe and visually observing and determining the amplified products by LFD. This technology does not require expensive equipment and can quickly and effectively amplify the target fragment under constant temperature. Importantly, the results of the amplified specimen can be visualized by the naked eye without additional devices. However, single RAA was not sensitive enough to detect low levels of target DNA [6]. Cas family members consist of Cas13, Cas12, and Cas14 effectors. Cutting the target nucleic acid can trigger the cleavage of irrelevant single-stranded DNA (ssDNA) or single-stranded RNA (ssRNA). This collateral cleavage has been applied to nucleic acid detection of different pathogens, including viruses, bacteria and parasitic protozoa [3, 8, 15]. CRISPR-Cas13a (formerly C2c2), belonging to Type VI Class 2 CRISPR-Cas effector, is a single-component enzyme targeting single-stranded RNA with a guide RNA. Binding with a complementary ssRNA will activate its activity of collateral ssRNA cleavage [1, 6]. In the specific high-sensitivity enzymatic reporter unlocking (SHERLOCK) platform, a quenched fluorophore is added to the collateral substrate, and the Cas13a system cleaves a nucleic acid reporter and generates a detectable signal, thus enabling target RNA detection [7]. CRISPR-Cas13a systems have been employed for nucleic acid detection of a variety of pathogens, including SARS-CoV-2, Zika virus, and dengue virus [6].

In this study, we applied RAA to detect the nucleic acid of *T. gondii* by amplifying target DNA, and then transcribed the DNA into RNA by T7 RNA polymerase promoter for subsequent LwCas13a detection, with the fluorescence signal being monitored by LFD visualization with the naked eye. A new type of RAA-LFD combined with the Cas13a detection method for *T. gondii* based on a 529 bp fragment was therefore successfully established. No cross-reaction with other parasites occurred, and the protocol is able to consistently detect 1 × 10^−6^ ng/μL of DNA per reaction. Amplification of a sample by the established RAA-Cas13a-LFD assay can be achieved at a constant temperature of 37 °C in less than 2 h.

The sensitivity of the RAA-Cas13a-LFD assay at a detection limit of 1 × 10^−6^ ng/μL per reaction is lower than that of qPCR. This different detection limit could be explained by the fact that different approaches can detect the lowest target concentration of *T. gondii* DNA in the samples [13]. Nonetheless, the RAA-LFD combined with Cas13a assay that we established in this work was still sensitive enough to permit timely detection of *T. gondii* in the blind test and evaluation of clinical blood samples. The positive and negative rates of RAA-Cas13a-LFD were consistent with those of qPCR for the 267 clinical blood samples. Four of the 267 clinical blood samples with unknown *T. gondii* infection were identified by qPCR and RAA-Cas13a-LFD, whereas only three cases out of the four positive specimens were ascertained by ECL (IgM^+^). These results also show that molecular techniques have certain advantages over specific antibody assays, such as ECL, in the diagnosis of disease in active and early infection phases, as the antibody test produces a result only after several weeks of parasitemia. The established RAA-Cas13a-LFD assay protocol was verified to be specific and sensitive in detecting *T. gondii*. Although qPCR and RAA-Cas13a-LFD are competent in different environment and laboratory conditions in auxiliary diagnosis of toxoplasmosis, our newly developed RAA-Cas13a-LFD protocol seems more rapid in procedure, offers easier visualization without complex and costly equipment, and importantly, it can be more suitable for on-site surveillance of *T. gondii*.

Ideal methods for detecting pathogens should be sensitive, specific, rapid, cost-effective and instrument-free [30]. *Toxoplasma gondii* was detected from clinical blood samples within 2 h and directly observed for the results by the naked eye under a constant temperature heating block in this study. Nevertheless, the detection is performed based on the premise that genomic DNA has been extracted from blood samples in advance. Since effective acquisition of nucleic acid detection is the basis of the simple and fast RAA-Cas13a-LFD assay in this current innovation, further development is needed, especially for a simple DNA acquisition procedure from the blood samples. An example would be the rapid and sensitive RAA assay to detect *Bordetella pertussis* using DNA obtained by boiling clinical samples of respiratory secretions, described in a previous study [34]. Furthermore, compared with qPCR, the detection limit of the blood-extracted *T. gondii* DNA by RAA-Cas13a-LFD assay in current study should achieve a more similar sensitivity to qPCR through screening of primer and probe combinations.

In summary, the rapid visual detection of *T. gondii* by combining recombinase polymerase amplification and lateral flow dipstick coupled with a CRISPR-Cas13a fluorescence assay that we have established could be superior to other molecular diagnostic tools in point-of-need application. This protocol is simple in operation, easier in visualization of the reaction results, and highly specific and sensitive, as well as promising for on-site testing of *T. gondii* infection in clinical settings.
